# Impact of Digital Intervention vs WHO Package of Essential Noncommunicable Disease Interventions Approach for Prevention and Control in Resource-Limited Settings: Protocol for a Quasi-Experimental Study

**DOI:** 10.2196/89021

**Published:** 2026-06-12

**Authors:** Mansi Gauniyal, Krishna Mohan Surapaneni, Richa Sinha, Nitish Mondal, Ashoo Grover, Bhavya Malhotra, Nikita Gupta, Upma Sharma, Ashish Joshi

**Affiliations:** 1 Foundation of Healthcare Technologies Society Delhi, Delhi India; 2 Panimalar Medical College Hospital & Research Institute Chennai, Tamil Nadu India; 3 Government Doon Medical College Dehradun, Uttarakhand India; 4 Department of Anthropology Sikkim University Gangtok, Sikkim India; 5 Indian Council of Medical Research Delhi, Delhi India; 6 School of Public Health, University of Memphis Memphis, TN United States

**Keywords:** noncommunicable diseases, digital health, WHO PEN, community-based intervention, health-seeking behavior, quasi-experimental study, India

## Abstract

**Background:**

Noncommunicable diseases (NCDs) are increasingly becoming a public health challenge, particularly in resource-limited settings, due to limited access to preventive care, early detection, and health literacy. Nevertheless, there is immense potential for digital technologies to enhance the overall community health. Similarly, the World Health Organization (WHO) Package of Essential NCD (PEN) interventions for primary health care in low-resource settings has demonstrated evidence of improving NCD outcomes. Nevertheless, its effectiveness in the Indian setting has not been explored.

**Objective:**

This study aims to evaluate the effectiveness of a mobile and web-based digital intervention compared with the WHO PEN approach and a no-intervention control group in improving health-seeking behaviors, risk factor modification, and disease management related to NCD prevention and control in resource-limited settings across 4 Indian sites.

**Methods:**

A quasi-experimental, mixed methods study will be conducted across 4 sites in India by using cluster-based allocation. The study will be conducted in 3 phases, where the insights gathered in the first phase will guide the development of a human-centered digital health intervention to help people nudge toward better health-seeking behaviors for common NCDs (diabetes or hypertension/cardiovascular diseases or both). The effectiveness of the digital intervention will be compared against the WHO PEN intervention and a control group that will receive no intervention. Standard validation tools will be used to assess behavior and changes related to modifiable risk factors. Data will be analyzed using descriptive and inferential statistics, with pre-post comparisons and between-group analyses. Qualitative data will be thematically analyzed to complement quantitative findings.

**Results:**

The funding for this study was received from the Indian Council of Medical Research in January 2025. In phase 1, across all sites, a total of 80 in-depth interviews (community and community health workers), 320 Knowledge, Attitude, And Practice questionnaires (community), and 32 focus group discussions with 320 individuals (community) were conducted from July 2025 to December 2025. This will be followed by data analysis, and findings will be used to guide the development of the digital intervention. The results of this study are expected to be published in 2028. This study is expected to demonstrate improved health-seeking behavior, self-management practices, and lifestyle modifications among participants exposed to the digital intervention compared with the WHO PEN and control groups. Findings will illuminate the feasibility and scalability of integrating digital health solutions within community-based NCD prevention frameworks in India.

**Conclusions:**

This protocol outlines a community-based, multi-site comparative study to evaluate the role of digital health interventions vis-à-vis WHO PEN in addressing NCD prevention and management. The results will contribute to evidence-based recommendations for strengthening digital health integration in resource-limited primary care settings.

**International Registered Report Identifier (IRRID):**

DERR1-10.2196/89021

## Introduction

### Background

Globally, noncommunicable diseases (NCDs) such as heart disease, diabetes, chronic respiratory problems, and cancer account for over 70% of deaths annually. Nearly 80% of these deaths happen in low- and middle-income countries [1]. In India, these diseases cause about 63% of all deaths, and the identified risk factors, such as limited or no physical activity, poor dietary habits, obesity, tobacco use, and alcohol consumption, are increasingly adding to the overall burden [2,3]. Despite several attempts in India to enhance basic health care with programs like the National Program for the Prevention and Control of NCDs, issues such as failure in early detection, treatment adherence, and receiving continuous care are still persistent in resource-limited settings [4].

The World Health Organization’s Package of Essential NCD (WHO PEN) interventions was introduced as a cost-effective strategy to enable primary health care workers to deliver standardized NCD services in low-resource environments [5]. The major focus of the WHO PEN protocol is on task shifting, screening at the early stage, and risk-based management by using simple tools and strategies. This intervention has been effectively executed to improve the management of diabetes and hypertension at the community level [6]. However, there are several limitations such as inconsistent implementation, limited health worker training, and weak follow-up systems that interfere with the long-term impact in real-world contexts [7].

### Digital Health and Its Potential in NCD Management

Recent years have witnessed an influx of digital health interventions that include mobile apps, web-based platforms, teleconsultations, and data-driven dashboards as a promising strategy to enhance health care access and continuity [8]. The use of mobile technologies for NCD prevention and control can enhance health literacy, modification in health-seeking behaviors, and remote monitoring, especially in resource-constrained settings [9]. Digital interventions have demonstrated potential in improving medication adherence, promoting physical activity, and supporting lifestyle modification [10,11].

In the Indian scenario, digital health has amplified to a significant level under the Ayushman Bharat Digital Mission, the goal of which is to integrate digital tools into primary health care delivery [12]. Despite all the developments achieved over the years, there has been limited evidence on the comparative effectiveness of digital interventions versus standardized models such as WHO PEN, particularly in diverse community settings. Furthermore, identifying and understanding the barriers and facilitators to technology adoption and contextual dissimilarities across geographic and sociodemographic groups is critical for scaling such interventions.

### Rationale for the Study

There has been an increasing utilization and acceptance of digital health platforms. However, there is a scarcity of evidence that compares and evaluates the impact of digital interventions relative to the established WHO PEN based standard care. Most existing studies focus on single disease domains such as diabetes or hypertension rather than comprehensive NCD prevention frameworks [13]. Moreover, multisite evidence capturing both urban and semiurban populations is scarce in India.

This proposed study will focus on diabetes, hypertension, or cardiovascular diseases. Further, this study will address the gaps by focusing on behavioral modification and implementing and evaluating the digital intervention developed as part of this study and the behavioral component of WHO PEN as part of a multi-site, quasi-experimental design comparing 3 groups:

1. A digital intervention group: This group will be provided with a digital intervention platform that will comprise multilingual educational modules to enhance health literacy, self-management techniques, self-care tracking, and digital follow-up modules designed to empower individuals and facilitate continuous engagement.
2. A WHO PEN group: This group will receive an intervention that would emphasize in-person screening, counselling, and follow-up based on established WHO guidelines.
3. A control group: This group will receive no structured intervention beyond the routine care that they receive as part of their daily routine.

### Study Significance

This study will provide robust empirical evidence on the comparative effectiveness of digital and WHO PEN interventions in enhancing the overall health of the affected individuals by nudging them toward better health-seeking behaviors and self-management practices. Identifying and understanding the barriers and facilitators that influence the adoption of digital interventions in community settings will serve as the foundational guide for developing barriers to digital intervention. Additionally, it will provide enough insights to guide the policy integration of digital health solutions in India’s existing NCD prevention and control programs. The mixed methods approach across 4 diverse sites, namely, Delhi, Chennai, Sikkim, and Dehradun, is expected to capture variations in the demographic, cultural, and infrastructural contexts, thereby enhancing the generalizability of the findings. This study should help by strengthening further discussion on the improvement of digital health and creating successful disease management plans in places with limited resources.

### Objectives

The primary objectives are as follows:

1. Identify influential factors shaping healthy behavior and design a tailored model for NCD (diabetes and cardiovascular diseases) prevention and self-management, targeting the urban slums in India.
2. Develop a personalized, human-centered, tailored, interactive, multilingual digital health intervention to enhance awareness of prevention and self-management of NCDs by using the findings of objective 1.
3. Evaluate the feasibility of developing a digital health intervention for NCD prevention or self-management in urban slums of India comparing it to WHO PEN interventions for primary care.

The secondary objectives are as follows:

1. Assess the changes in knowledge, attitude, and practice scores related to NCD prevention across the 3 study groups.
2. Identify contextual and sociodemographic factors that influence the adoption and sustained use of the digital platform, particularly in comparison to the implementation of WHO PEN.
3. Generate implementation insights and policy recommendations for integrating digital tools within India’s existing NCD prevention and control programs.

## Methods

### Study Design and Setting

This is a multi-site, quasi-experimental study, using a cluster-design sampling strategy. This study adopts a mixed methods approach to assess the efficacy of using the WHO PEN in comparison with a control group that received no intervention and a digital intervention platform. The study is being conducted in urban poor settings in India across 4 sites with differing geographic and sociocultural characteristics. Allocation to study arms will occur at the community (cluster) level. Within each site, community clusters (wards/slum clusters) will be assigned to one of the 3 intervention arms to reduce contamination between participants and to align with local service delivery structures. All eligible individuals within a cluster are enrolled into the arm designated for that cluster.

The study will span 3 years and execute 3 distinct phases to achieve the 3 stated objectives. The interventions will be deployed in the third phase of the study and will include follow-up evaluations at 3 months, 6 months, 9 months, and 12 months post intervention.

### Inclusion and Exclusion Criteria

The individuals will be included if they are 18-70 years old, have diabetes or hypertension or cardiovascular diseases and/or both, can provide written informed consent, have access to a mobile phone (Android or any smartphone), and are willing to use technology. They will be excluded if they are younger than 18 years, have a terminal illness, have impaired cognition, are pregnant, fail to adhere to study procedures, or they are currently enrolled in another interventional study addressing the same outcomes.

### Data Collection and Sampling Strategy

To ensure that each site implements all 3 arms while preserving feasibility and minimizing contamination, this study will use a clustered quasi-experimental design at the local community level.

#### Objective 1

To meet objective 1, both the quantitative and qualitative data will be collected. At each site, 8 focus group discussions will be conducted in community settings, with 8-10 individuals participating in each group. The in-depth interviews will be conducted with 10 individuals from the community and 10 community health workers. The Knowledge, Attitude, and Practice questionnaire, along with the other validated assessment tools, will be administered to 80 individuals from each site.

#### Objective 2

To meet objective 2, a total of 80 participants will be enrolled across 4 zones at each site to obtain their feedback on the developed digital platform.

#### Objective 3

To meet objective 3, participants at each site will be assigned to one of the following 3 study arms based on the site’s designated intervention:

- Digital intervention arm: Participants will receive access to a mobile and web-based digital health platform developed by the Foundations of Healthcare Technologies Society, designed to promote NCD screening, health education, and behavior modification through interactive modules, personalized reminders, and self-tracking tools.
- WHO PEN arm: Participants will receive structured screening, counselling, and management services as per the WHO PEN protocols 1 and 2, implemented through trained health care providers and community health workers.
- Control arm: Participants will receive only routine health services available in their local setting, without any structured digital or WHO PEN-based intervention.

The sample size was determined to detect differences in primary outcomes (health-seeking behavior and NCD self-management) between the 3 arms. The initial sample size was adjusted for the cluster design, considering Cohen *d* as 0.30 for a small to moderate effect size, with 80% power and a significance level of 5%. Each study site will include 16 community clusters, resulting in a total of 64 clusters across the 4 sites. From each cluster, nearly 30 participants will be included with intracluster correlation coefficient of 0.02 and design effect of 1.58. The sample size will be adjusted for 15%-20% of attrition rate during the 12 months of follow-up period, resulting in the total sample size of 2400 (600 per site).

**Figure 1:**
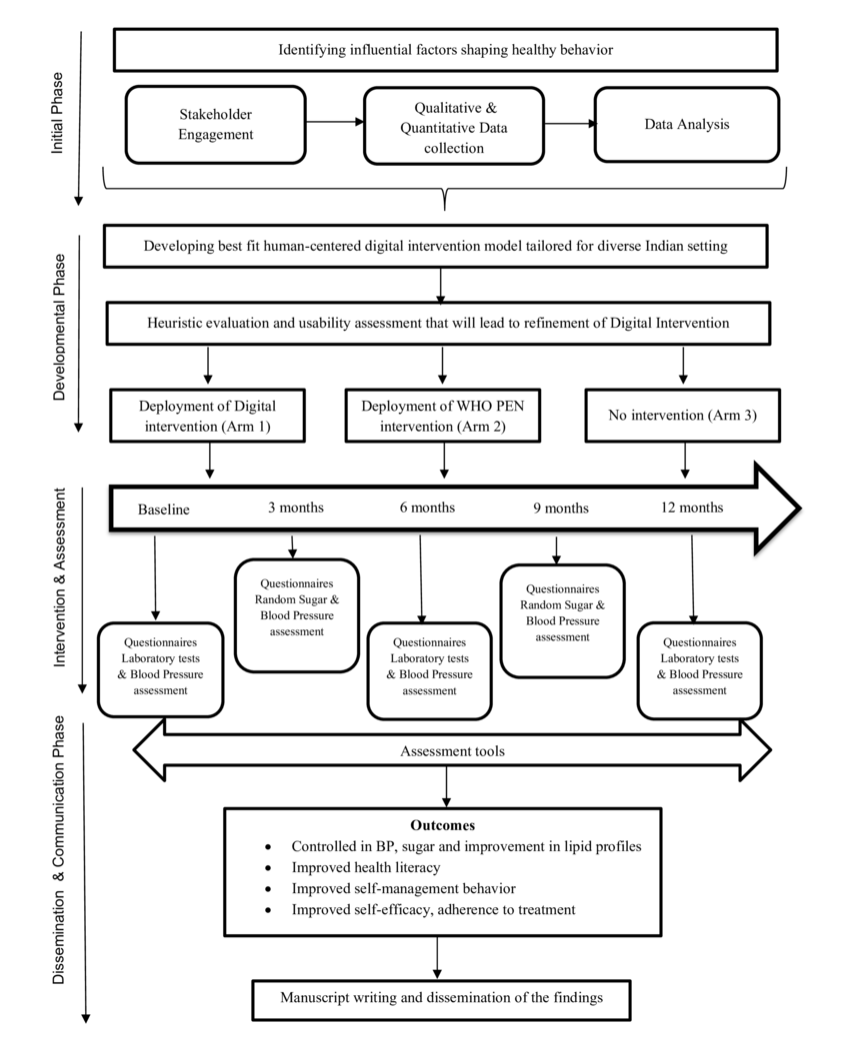
The study implementation flowchart. BP: blood pressure; WHO PEN: World Health Organization Package of Essential Noncommunicable Disease Interventions.

The comparative evaluation across these 3 groups will reveal differential effects on health-seeking behavior, self-management practices, and clinical outcomes. Figure 1 illustrates the study design framework, detailing the phases of data collection and intervention implementation. The summary of the quantitative data collection tools and outcome measures in the third phase of the study are described in Table 1.

**Table 1:** Summary of the quantitative data collection tools and outcome measures in the third phase.

Tool/instrument	Purpose/domain	Administration time points^a^
Sociodemographic and Health Questionnaire	Background characteristics	T_0_
Anthropometry and Clinical Tests	Physical and biochemical outcomes	T_0_, T_1_, T_2_, T_3_, T_4_
Knowledge, Attitude, and Practice Questionnaire	Knowledge, attitude, practice	T_0_, T_2_
Global Physical Activity Questionnaire	Physical activity	T_0_, T_1_, T_2_, T_3_, T_4_
24-hr Dietary Recall	Dietary habits	T_0_, T_1_, T_2_, T_3_, T_4_
Alcohol Use Disorders Identification Test	Alcohol use	T_0_, T_1_, T_2_, T_3_, T_4_
Perceived Stress Scale-10 items	Perceived stress	T_0_, T_1_, T_2_, T_3_, T_4_
Pittsburgh Sleep Quality Index	Sleep quality	T_0_, T_1_, T_2_, T_3_, T_4_
Summary of Diabetes Self-Care Activities	Diabetes self-care	T_0_, T_1_, T_2_, T_3_, T_4_
Medication Compliance Questionnaire	Medication adherence	T_0_, T_1_, T_2_, T_3_, T_4_
System Usability Scale	Usability of the digital platform	T_0_, T_1_, T_2_, T_3_, T_4_
Laboratory tests	Physical and biochemical outcomes (HbA_1c_, fasting sugar, lipid profile)	T_0_, T_2_, T_4_

^a^T_0_: baseline; T_1_: third month; T_2_: sixth month; T_3_: ninth month; T_4_: twelfth month.

### Data Collection Tools

#### Qualitative Tools

##### In-Depth Interviews

In-depth interviews will be conducted with participants from community settings and community health workers to explore their experiences, perceptions, and the barriers or facilitators related to the digital interventions and the WHO PEN program.

##### Focus Group Discussions

Focus group discussions will be conducted with community members at each site to understand their collective perspectives on NCD prevention and digital engagement.

#### Quantitative Data Collection Tools

##### Sociodemographic Characteristics

Information was recorded on age, gender, marital status, educational qualification, occupation, and monthly household income (INR).

##### Anthropometric and Clinical Assessments

Data on anthropometric measurements (height, weight, and waist circumference) will be taken. In addition, blood pressure levels will be recorded using a digital sphygmomanometer, and random blood glucose levels will be recorded using a glucometer. Additionally, the third phase of the study will include laboratory tests for fasting blood sugar and lipid profiles at baseline and follow-up at 3 months, 6 months, 9 months, and 12 months.

##### Familiarity With the Use of Technology

Data will be gathered on the usage of mobile phones and the ability of the participants to read and send text messages, along with access to the internet.

##### NCD-Related Knowledge, Attitude, and Practices

This will be assessed using a series of questions about individuals' existing disease-related knowledge and their attitudes and practices toward its self-management.

##### 24-Hour Dietary Recall

A 24-hour dietary recall will be conducted to ascertain the dietary intake of the enrolled participants. The nutrient consumption within the last 24 hours will be computed for macronutrients and micronutrients, that is, energy, proteins, carbohydrates, total fiber, fat, total saturated fatty acids, total monounsaturated fatty acids, total polyunsaturated fatty acids, retinol, and vitamin E [14].

##### Physical Activity

This will be assessed using the Global Physical Activity Questionnaire. MET-minutes (metabolic equivalent of task) are calculated using the formula: Total MET-minutes=(minutes per day × MET value) × days per week [15].

##### Perceived Stress Scale

The Perceived Stress Scale is the most widely used psychological instrument originally developed for measuring perceived stress [16]. It is a measure of the degree to which situations are appraised as stressful. The Perceived Stress Scale is a 10-item test that rates the frequency of stress-related experiences on a 5-point scale from 0 (never) to 4 (very often).

##### Pittsburgh Sleep Quality Index

The Pittsburgh Sleep Quality Index is a standardized self-rated questionnaire to assess sleep quality and disturbances over a 1-month time interval [17].

##### Medication Compliance Questionnaire

Medication adherence will be assessed using a Medication Compliance Questionnaire that consists of 7 questions. A 4-point Likert scale will be used for rating each question (1=all the time, 2=most of the time, 3=sometimes, 4=none of the time), with higher scores indicating better adherence [18].

##### Summary of Diabetes Self-Care Activities

This tool will be used to examine adherence to diet, exercise, medication, and glucose monitoring among participants with diabetes [19].

##### Alcohol Use Disorders Identification Test

This tool will be used to assess alcohol consumption, drinking behaviors, and alcohol-related problems among the participants. It is a 10-item screening tool developed by the WHO that helps to identify individuals with hazardous or harmful patterns of alcohol use in both clinical and community settings [20].

##### System Usability Scale

The System Usability Scale will be used to assess the usability of the developed digital intervention. The System Usability Scale, a widely accepted 10-item tool, measures the usability of any given system or product [21].

### Data Analysis Plan

Interviews and focus groups will be audio-recorded, transcribed verbatim, and translated to English where necessary. A thematic analysis approach will be applied using NVivo software (Lumivero). Triangulation across all the data sources will enhance validity. Data will be analyzed using SPSS (version 29; IBM Corp) and R software (R Foundation for Statistical Computing). Heuristic evaluation will involve the estimation of severity ratings, frequency of usability issues, time taken to complete tasks, and interrater reliability. Descriptive statistics will include means (SDs), medians (IQRs), and proportions to summarize baseline characteristics. Between interventions and control arms, the analysis would be performed by ANCOVA or generalized linear models, adjusting for baseline values, cluster effects, and site-level covariates. Statistical significance will be set at *P*<.05, with 95% CIs.

A generalized linear mixed-effects model will be used to conduct the primary analysis, because the data are clustered and longitudinal in nature. The generalized linear mixed-effects model will include random intercepts for the clusters of communities and the individual study participants to account for the intracluster correlation and repeated measurements over time. Intervention arm, time, and their interaction will be modelled as fixed effects, with study site included as a fixed effect to control for contextual differences. To control the baseline imbalance in outcome variable values, the baseline value will be used as a covariate in the analysis. The maximum likelihood estimation within the mixed-effects framework will handle the missing data in the study, with sensitivity analyses using multiple imputation. Pairwise comparisons among the 3 study arms will be conducted. This will be adjusted for multiple testing using the Holm-Bonferroni method, with statistical significance assessed at 2-tailed α level of .05.

### Project Timelines

The project duration will be 3 years, from January 2025 to January 2028. A comprehensive overview of the timelines of the different phases of the project is provided in Table 2.

**Table 2:** Scheduled timeline of the tasks in the study.

Tasks		Months											
		1	2-3		4-6		7-14	15-20^a^			32-33	34-36	
Phase I													
	Literature review, designing, and planning of the study, and stakeholder meeting	✓											
	Qualitative data (in-depth interviews/focus group discussions) and quantitative data collection (Knowledge, Attitude, and Practice)	✓	✓	✓									
	Analysis of mixed data		✓	✓									
Phase II													
	Development intervention					✓							
	Heuristic evaluation					✓							
	Usability of the developed digital intervention system					✓							
	Refinement					✓							
Phase III													
	Final deployment of the proposed digital and WHO PEN^b^ intervention								✓				
	Study recruitment								✓				
	Baseline and follow-up data collection								✓				
	Data preparation and statistical analysis									✓			
	Report writing and manuscript preparation									✓			✓
	Dissemination of research findings												✓

^a^Follow-up until week 32.

^b^WHO PEN: World Health Organization Package of Essential Noncommunicable Diseases.

### Quality Assurance

Quality will be ensured across all the elements of data collection and data handling. Data collection and entry will be performed by well-trained field staff. Standard operating procedures will be prepared and followed for qualitative and quantitative data collection. Training sessions will be conducted for all staff involved to orient them to the study aims, objectives, study area, inclusion and exclusion criteria, procedures for obtaining informed consent, and the standard operating procedures for the data collection tools. Weekly meetings and continued training sessions will be an integral part of the project to ensure high quality.

### Ethical Considerations

Ethical approval has already been obtained from the institutional human ethics committees of all participating sites from Delhi, Dehradun, Sikkim, and Chennai.

- Delhi: The Foundations of Healthcare Technologies Society obtained its ethical approval in December 2024 with the approval number FHTS/IHEC/2024/01.
- Dehradun: Government Doon Medical College obtained its ethical approval in June 2025, with the approval number GDMC/IEC/2025/12.
- Sikkim: Sikkim University obtained its ethical approval in June 2025, with the approval number SU/REG/F-1/03/2019/Vol-II/771.
- Chennai: Panimalar Medical College Hospital and Research Institute obtained its ethical approval in February 2025, with approval number PMCHRI-IHEC-286.

Written informed consent will be obtained from the selected participants in the preferred local language at all 4 sites. This study will strictly protect the data confidentiality and anonymity of research participants. The study will comply with the principles of the Declaration of Helsinki (2013) and the Indian Council of Medical Research ethical guidelines.

### Expected Outcomes

The proposed study will offer conclusions about the relative effectiveness and feasibility of digital interventions compared to WHO PEN approaches and identify tailored strategies for NCD prevention and control in diverse resource-limited settings. It will assist in developing an intervention model that integrates strategies specifically designed for urban slums or areas, addressing the unique challenges they encounter. In addition to behavioral and self-management outcomes, clinical assessments of fasting blood sugar, lipid profile, and blood pressure will be conducted to evaluate changes in metabolic risk factors across all 3 study groups. These objective measures will provide evidence of physiological improvements associated with the intervention and help validate its effectiveness in reducing NCD risks. This research could help individuals to enhance their health-seeking behavior, manage their disease condition effectively, and ease the load of certain common NCDs in resource-limited settings by helping people make better health-related choices.

## Results

The funding for this study was received from the Indian Council of Medical Research in January 2025. In phase 1, across all sites, a total of 80 in-depth interviews (community and community health workers), 320 Knowledge, Attitude, And Practice questionnaires (community), and 32 focus group discussions with 320 individuals (community) were conducted from July 2025 to December 2025. This will be followed by data analysis, and findings will be used to guide the development of the digital intervention. The final results of this study are expected to be published in 2028. This study is expected to demonstrate improved health-seeking behavior, self-management practices, and lifestyle modifications among participants exposed to the digital intervention compared with the WHO PEN and control groups. Findings will illuminate the feasibility and scalability of integrating digital health solutions within community-based NCD prevention frameworks in India.

## Discussion

### Overview

The burden of NCDs falls disproportionately on low- and middle-income countries, where cardiovascular disease, cancers, chronic respiratory disease, and diabetes drive 80% of premature deaths, posing a serious threat to global health and socioeconomic development [1,9]. In India, socioeconomically disadvantaged groups, who lack timely access to health care, bear the brunt of high out-of-pocket costs and are particularly vulnerable to the situation. The rapid adaptation of urban lifestyles, along with the widespread acceptance of nonactive lifestyles, unhealthy eating habits, and addictive behavior, has exacerbated the problem of NCDs’ burden [6,10-12]. The lack of adequate health care infrastructure, financial limitations, and lack of awareness make it extremely difficult for vulnerable individuals in low- and middle-income countries to access timely health care. Inadequate diagnosis or management of NCDs results in serious health problems, diminished productivity, and crippling medical expenses [10]. To overcome this barrier, there is an urgent need for low-cost, scalable interventions, such as digital platforms, to enable communities at risk to maintain self-care and overcome the accessibility gap [22].

The use of digital interventions is gaining traction as a way to enhance primary health care while reducing the burden of NCDs. It is becoming increasingly apparent that digital interventions are improving primary health care and reducing the burden of NCDs. The digital approaches facilitate real-time, adaptable, and tailored communications through mobile platforms, which are increasingly adopted by a wide range of ages and literacy levels [12]. By providing personalized, real-time feedback and sustained engagement, smartphone apps, text messages, and web-based tools improved dietary habits, exercise, and smoking cessation [22-24]. Studies have reported that digital health interventions can improve awareness, behavioral change, and self-management of NCDs in economically disadvantaged countries [25].

Thus, this protocol outlines a novel, multi-site, quasi-experimental study designed to compare the effectiveness of a human-centered digital health intervention against the WHO PEN approach in resource-limited settings. This study addresses that gap by implementing both interventions concurrently across diverse and contextually relevant geographic and sociodemographic settings in India, thereby ensuring that the digital solution is grounded in user needs, cultural context, and practical feasibility. This protocol sets the stage for digital transformation in primary NCD care, closely following India's National Digital Health Mission and the global Universal Health Coverage.

### Strengths and Potential Limitations

A mixed methods approach will be utilized, integrating insights from behavioral, clinical, and qualitative measures to capture multidimensional outcomes. The human-centered design will ensure the digital intervention is tailored to the context of the target population, enhancing relevance and user engagement. The inclusion of geographically and culturally diverse sites such as Delhi (plains), Dehradun (valley), Chennai (coastal), and Sikkim (hilly) would provide an additional contextual understanding that would support external validity. Moreover, the incorporation of digital monitoring systems facilitates real-time data acquisition and guarantees the integrity of the interventions.

However, this study anticipates certain limitations. The nonrandomized allocation of participants may introduce selection bias, although cluster-based assignment and analytical adjustments will be employed to minimize its effects. Additionally, variations in digital literacy among participants may influence engagement with the intervention. Despite these constraints, the study is expected to yield robust insights into the differential effectiveness, user experience, and implementation feasibility of digital solutions for NCD prevention within the low- and middle-income country-setting contexts.

## Data Availability

The data supporting this study’s findings are available upon request from the corresponding author.

## Appendix

Multimedia Appendix 1 Peer review report by the Indian Council of Medical Research.

## Abbreviations

NCD: noncommunicable disease
WHO: World Health Organization
WHO PEN: World Health Organization Package of Essential Noncommunicable Disease Interventions
