# Supplementary material for: Impact of Digital Intervention vs WHO Package of Essential Noncommunicable Disease Interventions Approach for Prevention and Control in Resource-Limited Settings: Protocol for a Quasi-Experimental Study
Source: JMIR Res Protoc. 2026 Jun 12;15:e89021. doi: 10.2196/89021 (PMC13309768; doi:10.2196/89021)
Supplement: Multimedia Appendix 1 [file resprot_v15i1e89021_app1.pdf]

Gmail

Mansi Gauniyal <mansigauniyal@gmail.com>

ICMR: Proposal Id IIRPIG-2024-01-01632 result (no-reply to this email)

no-reply@hq.icmr.org.in <no-reply@hq.icmr.org.in>
To: Dr Mansi Gauniyal <mansigauniyal@gmail.com>
Cc: ICMR-ePMS <po.epms@icmr.gov.in>, ICMR-ePMS <no-reply@hq.icmr.org.in>

22 August 2024 at 17:28

Dear Dr Mansi Gauniyal,

This is with reference to your proposal Id "**IIRPIG-2024-01-01632**", titled "**Evaluating the Impact of Digital Intervention versus WHO PEN Approach in Non-Communicable Disease Prevention and Control in Resource Limited Settings: A Quasi - Experimental Study**".

Your project is under consideration for funding. Final decision will depend on how well you respond to the technical concerns, mentioned below:

Technical Comments:

Methodology
1. Justification related to study design as to why is it quasi-experimental and not experimental.
Implementation strategy
1. There are concerns about the implementation and delivery of the interventions. Specifically, it is not clear who will deliver the interventions and how the rollout will be managed.
2. The proposal should define the roles of frontline health workers, to ensure effective implementation and address potential challenges related to the acceptance and usage of the digital tools by the target population.

Please address comments within two weeks of receipt of this email and submit your revised proposal. For ease of review, highlight the revisions and provide a separate document with point by point justifications. You may please attach appropriate information/ publication/ preliminary data with your comments in one single pdf.

Please address comments within two weeks of receipt of this email and submit your revised proposal. If we do not receive your proposal within the given time, your proposal will be removed from the list of proposals under consideration.

For any clarification/s, you may contact "Dr. Saurabh Sharma (sharmas@icmr.gov.in)"

Important Note: This is an automated email. Kindly refrain from replying to this email.

Warm Regards
Program Officer

icmr
INDIAN COUNCIL OF MEDICAL RESEARCH
Serving the nation since 1911

e-Project Management System
Indian Council of Medical Research
Department of Health Research
Ministry of Health & Family Welfare
Government of India
Ansari Nagar, New Delhi - 110029, India
www.icmr.gov.in

</html

Gmail **Mansi Gauniyal <mansigauniyal@gmail.com>**

Regarding Codal documents to be submitted to ICMR with reference to the Project ID: "IIRPIG-2024-01-01632"

Saurabh Sharma <sharmas@icmr.gov.in> 23 October 2024 at 11:05
To: mansigauniyal <mansigauniyal@gmail.com>

Dear Dr.Mansi Guniyal,

We are pleased to inform you that your project titled "Evaluating the Impact of Digital Intervention versus WHO PEN Approach in Non-Communicable Disease Prevention and Control in Resource Limited Settings: A Quasi - Experimental Study"; **IIRPIG-2024-01-01632**, has been selected for funding. Please submit the following documents for completion of the codal formalities. Please also submit the revised proposal through the epms portal.

1. Institutional Ethical Clearance
2. Budget justification of staff, contingency, travel etc. - (Format attached, Annex IV)
3. Declaration & Attestation - (Format attached, Annex V)
4. Acceptance Form - (Format attached, Annex VII)
5. Bank and Account Details with a cancelled cheque copy - (Format attached, Annex VIII) (Only Saving Account)
6. Plagiarism Undertaking - (Format attached, Annex IX)
7. Department of Scientific & Industrial Research (DSIR) certificate (if any govt. organization is exempted from the DSIR certificate then the exemption certificate duly signed by the Head of the Institute is to be provided)
8. CV of PIs and Co-PIs in the attached ICMR format.

The formats for codal documents are attached below for your reference.

Kindly submit the documents at the earliest.

Please feel free to communicate through this email, if any further clarifications are required.

warm regards

Dr. Saurabh Sharma,
MBBS, MD (Community Medicine),PGDMRCH
Scientist-D,
Delivery Research Division,
Indian Council of Medical Research,
Ansari Nagar, New Delhi, 110029
Mob No - +91 - 7840870009
